# Optimal bandwidth selection in stochastic regression of Bio-FET measurements

**DOI:** 10.1007/s00285-025-02231-y

**Published:** 2025-06-10

**Authors:** Luis A. Melara, Ryan M. Evans, Seulki Cho, Arvind Balijepalli, Anthony J. Kearsley

**Affiliations:** 1 Department of Mathematics, Shippensburg University of Pennsylvania, 1871 Old Main Drive, Shippensburg, 17257 PA USA; 2 Applied and Computational Mathematics Division, National Institute of Standards and Technology, 100 Bureau Drive, Gaithersburg, 20899 MD USA; 3 Chemical and Biomolecular Engineering, University of Maryland, College Park, 20742 MD USA; 4 Microsystems and Nanotechnology Division, National Institute of Standards and Technology, 100 Bureau Drive, Gaithersburg, 20899 MD USA

**Keywords:** Stochastic differential equation, Stochastic regression, Biological field effect transistors, 60H10, 62L20, 62M10, 92-10

## Abstract

Biological field effect transistors (Bio-FETs) are modern bioelectronics instruments that offer rapid, low-cost and accurate point of care (POC) biomarker measurements. The time series data produced by these devices contain noise which interferes with quantitative analysis. Stochastic regression, which relies on modeling the measurement with a linear stochastic drift-diffusion equation with unknown coefficients, is employed to separate signal from noise. Coefficients are estimated through local weighted regression and maximum likelihood estimation, both of which depend on a kernel function and the size of a bandwidth parameter. In this work we determine the optimal bandwidth parameter associated with an experimental Bio-FET measurement, by considering three distinct but related kernel functions. Cross validation is performed with respect to different instrument aspect ratios. Results show optimal bandwidth parameters are surprisingly consistent across aspect ratios, and suggest a choice of kernel function.

## Introduction

New point of care (POC) medical diagnostic instruments promise to fundamentally improve healthcare delivery by giving physicians an accessible way to measure biomarkers and thus make timely diagnostic decisions. Dire need for such technologies was illustrated by the recent COVID-19 pandemic wherein existing POC diagnostic tests proved less accurate than those based on quantitative polymerase chain reaction (Patrone et al. 2020). The latter requires significant sample preparation, specialized facilities and trained personnel. For this reason, test results are often reported days after being administered. The need for instruments that facilitate quality accessible care has led to an intense interest in their research and development.

**Fig. 1:**
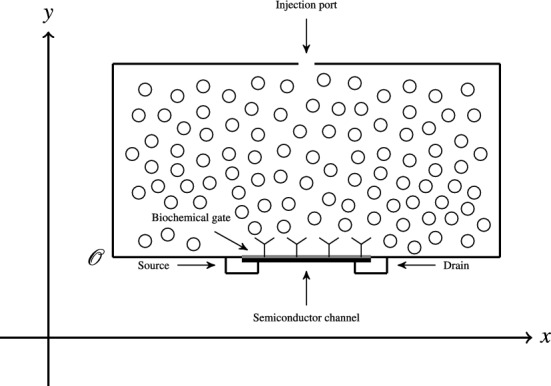
Idealized cross-sectional schematic of a Bio-FET experiment. Chemical reactants injected at the top of the instrument diffuse to bind with receptors confined to the biochemical gate. In our schematic, chemical reactants in solution are represented as circles spread throughout the instrument, and receptor sites are depicted with Y’s on the biochemical gate. This schematic is not to scale. The orientation of the *x* and *y* axes have been shown on the left, along with the location of the origin ${\mathcal {O}}$

**Fig. 2:**
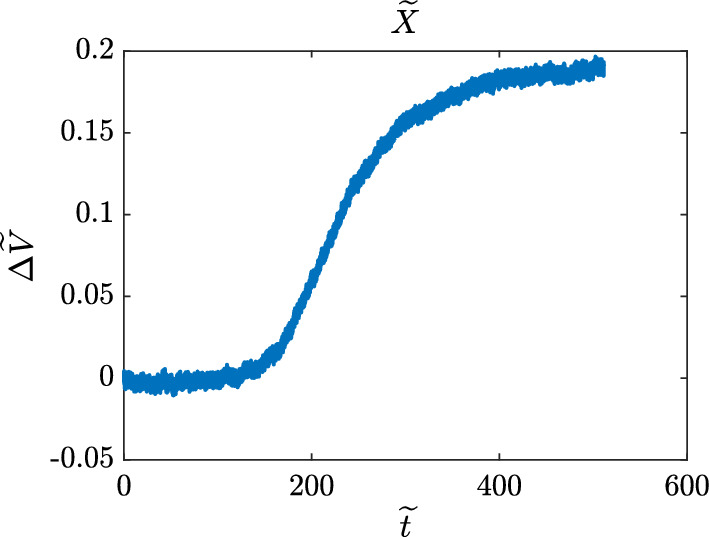
Experimental Bio-FET signal. Streptavidin injected into the solution-well of a Bio-FET has diffused through the solution-well onto the biochemical gate to bind with immobilized biotin, creating the displayed signal. More information can be found in (Evans et al. 2020)

Biological field effect transistors (Bio-FETs) are modern bio-electronics instruments that show particular promise, since they are low-cost and portable tools that yield highly precise measurements on a rapid time scale. The dimensions of these azimuthally symmetric instruments are on the order of millimeters. An idealized cross-sectional schematic is shown in Fig. 1. During a typical experiment, chemical reactants injected through a port at the top of the solution-well diffuse through the well to bind with receptors immobilized to the biochemical gate, which together with the semiconductor translate binding of chemical reactants into a signal, an example of which is shown in Fig. 2.

Figure 2 clearly reveals noise that can adversely affects quantitative analysis. Stochastic regression is a denoising approach that has successfully been applied to mass spectrometry (Kearsley et al. 2014) where a signal is modeled with a linear stochastic drift-diffusion equation that contains unknown coefficients. These coefficients are estimated through local weighted regression and maximum likelihood estimation, both of which depend on the local averaging function and the size of the local averaging window. Selection of the local averaging function, referred to as the *kernel function*, and the local averaging window size parameter or *bandwidth parameter* control the performance of the method. In this paper the optimal bandwidth parameter associated with an experimental Bio-FET measurement is calculated by considering three related kernel functions. We begin in Sect. 2 by describing our drift-diffusion model and outlining our regression procedure. In Sect. 3 three optimality measures are considered and employed to optimally reconstruct signal from Bio-FET measurement data. Cross validation of optimal bandwidths is described in Sect. 4 with different synthetic measurements corresponding to different aspect ratios $\epsilon$, the ratio of the solution-well’s height to the biochemical gate diameter. Concluding remarks will be given in Sect. 5.

## Regression Model

Our drift-diffusion model for the signal $X_t$ takes the form:

2.1a $$\begin{aligned}&\textrm{d}X_t=M (t,X_t)\ \textrm{d}t+\Sigma (t,X_t)\ \textrm{d}W_t, \end{aligned}$$

2.1b $$\begin{aligned}&X_t(0)=X_0, \end{aligned}$$

where $M (t,\ X_t)$ and $\Sigma (t,\ X_t)$ are respectively the drift and diffusion coefficients, and $W_t$ is a standard Brownian motion term with independent and normally-distributed increments such that

2.2a $$\begin{aligned}&\textrm{E}[\textrm{d}W_t]=0, \end{aligned}$$

2.2b $$\begin{aligned}&\textrm{Var}[\textrm{d}W_t]= \textrm{d}t. \end{aligned}$$

In this work we assume that (2.1) holds on a finite interval $[0,\ T]$, and from (2.1) it follows that we are also assuming that noise is additive and normally-distributed; no assumptions are made on the autocorrelation. Each of the independent and dependent variables in (2.1) are dimensionless, and may readily be cast in dimensional form by introducing unit characteristic scales for time $\widetilde{t}_{\textrm{c}}$ and the signal $\widetilde{X}_{\textrm{c}}$ and setting

2.3a $$\begin{aligned}&\widetilde{t} := \widetilde{t}_{\textrm{c}}\ t, \end{aligned}$$

2.3b $$\begin{aligned}&\widetilde{X}_{\widetilde{t}} := \widetilde{X}_{\textrm{c}}\ X_t. \end{aligned}$$

We work with the dimensionless formulation (2.1) and convert to dimensional variables only when discussing solutions of (2.1), for ease of comparison with physical experiments.

Our model (2.1) is a drift-diffusion equation requiring the drift and diffusion coefficients to be specified. We take the drift term to be a linear function of $X_t$

2.4 $$\begin{aligned} M(t,X_t)=a_0(t)+a_1(t)X_t, \end{aligned}$$

where $a_0(t)$ and $a_1(t)$ are functions to be determined. There are many possible forms for the diffusion coefficient, among them are

2.5a $$\begin{aligned}&\sigma (t,X_t)=b(t), \end{aligned}$$

2.5b $$\begin{aligned}&\sigma (t,X_t)=b(t)X_t, \end{aligned}$$

2.5c $$\begin{aligned}&\sigma (t,X_t)=b_0(t)X_t^{b_1(t)}, \end{aligned}$$

where *b*(*t*), $b_0(t)$, and $b_1(t)$ are functions to be determined. We shall comment on each of the choices in (2.5); first considering (2.5b), we observe that this choice renders (2.1) singular if the data are continuous and not strictly positive or negative. Similar problems may arise with (2.5c) if $\lim _{t \rightarrow t_i}X_t=0$ and $b_1(t)$ does not approach zero at the appropriate rate. Furthermore (2.5c) will result in non-real coefficients if for some $t_i$ during [0, *T*] the datum $X_{t_i}$ is negative and $b_1(t_i)$ assumes a non-integral value. These difficulties could ostensibly be overcome by mapping the data $X_{t_i}$ to $X_{t_i}+c$ for some constant *c*, but we nevertheless prefer the choice (2.5a) for which there are no restrictions.

Now if we had the exact coefficients we could partition the interval $[0,\ T]$ into *M* subintervals with a set of nodes $t_0<t_1<\cdots <t_M$ and apply an Euler-Maruyama discretization of the form

2.6a $$\begin{aligned}&\Delta X_{i}=[a_0(t_i)+a_1(t_i)]\Delta t+b(i)\Delta W_{i},\end{aligned}$$

2.6b $$\begin{aligned}&X_0=X_t|_{t=0}, \end{aligned}$$

for $i=0,\ \ldots ,\ M-1$. In (2.6) we have let $\Delta t=t_{i+1}-t_i$, $\Delta X_{i}=X_{i+1}-X_{i}$, and $\Delta W_{i}=W_{i+1}-W_{i}$, where $X_i$ and $W_i$ are discrete approximations to their continuous counterparts $X_{t_i}$ and $W_{t_i}$. From the properties in (2.2) it follows that $\Delta W_i\sim {\mathcal {N}}(0,\ \Delta t)$. Formula (2.6) suggests that we can recover the true signal through the difference equation

2.7a $$\begin{aligned}&\Delta X^{(\textrm{d})}_{i}=[a_0(t_i)+a_1(t_i)]\Delta t_i,\end{aligned}$$

2.7b $$\begin{aligned}&X^{(\textrm{d})}_0=X_t|_{t=0}, \end{aligned}$$

for $i=0,\ \ldots ,\ M-1$. In (2.7) we have used have used $X^{(\textrm{d})}$ to denote the deterministic component of the signal. We could recover the stochastic component through the formula

2.8 $$\begin{aligned} X^{(\textrm{w})}_i = b(t_i)\ \Delta W_i, \end{aligned}$$

for $i=1,\ \ldots ,\ M$, since our initial condition is exact.

Although we do not have the exact form of $a_0(t),\ a_1(t),$ or *b*(*t*), following (Kearsley et al. 2014) we can estimate $a_0(t)$ and $a_1(t)$ through local weighted regression and *b*(*t*) through maximum-likelihood estimation. To this end we assume that the drift and diffusion coefficients are well-approximated by constants over a set of narrow windows

2.9 $$\begin{aligned} (t_j-h,\ t_j+h), \end{aligned}$$

where each one of the nodes $t_j$ is chosen from the set $\{t_i \}_{i=1}^M$. The parameter *h* in (2.9) is the *bandwidth parameter*, and in this work we take it to be an integral multiple *k* of the time step

2.10 $$\begin{aligned} h= \Delta t\, k. \end{aligned}$$

We note that we may not be able to evenly partition the interval [0, *T*] into subintervals of the form (2.9); suppose this is the case, that we cannot fully partition the interval $[0,\ T]$ into subintervals of the form (2.9) and that there are *l* such subintervals in total. From (2.9) and (2.10) it follows that $(t_k-h,\ t_k+h)$ will be the first subinterval to lie in the interior of $[0,\ T]$, and that the remaining $l-1$ will take the form

2.11 $$\begin{aligned} (t_{k+j(2k)}-h, t_{k+j(2k)}) \end{aligned}$$

for $j=1,\ \ldots ,\ l-1$. There will also be points in a neighborhood of the right boundary of $[0,\ T]$ that do not fall within any of the aforementioned *l* subintervals, and it is the case that the procedure described below for estimating the drift and diffusion coefficients applies only to nodes that lie within one of the *l* subintervals of the form (2.11) for $j=0,\ \ldots ,\ l-1$. To estimate drift and diffusion coefficients for points near the right boundary that do not fall within any of these *l* subtintervals, we linearly extrapolate the data to *r* fictitious points, where *r* is the least positive integer such that 2*k* divides $M+r$. Then we apply the procedure described below to estimate the drift and diffusion coefficients in that last extended subinterval, and discard the fictitious points when finished, since they are unnecessary for time stepping. This extrapolation procedure is justified for the signals herein since data points $(t_i,\ X_{t_i})$ for which $t_i$ is close to the terminal time *T* are in a steady regime. In general, our extrapolation procedure is expected to work for signals that have the property that data points $(t_i,\ X_{t_i})$ for which $t_i$ is close *T* exhibit linear behavior.

Turning our attention to the drift coefficients, we take their approximation to be the solution to the local weighted regression problem

2.12 $$\begin{aligned} \{\widehat{a}_0(i),\ \widehat{a}_1(i)\}=\textrm{argmin}_{a_0(t_i),\ a_1(t_i)}\sum _{j=0}^{M-1} \left( \frac{\Delta X_j}{\Delta t}-a_0(t_i)-a_1(t_i)X_j\right) ^2 K_h\left( \frac{t_j-t_i}{h}\right) . \end{aligned}$$

In (2.12) we have let $\widehat{a}_0(i)$ and $\widehat{a}_1(i)$ denote approximations to the their continuous counterparts $a_0(t_i)$ and $a_1(t_i)$. Here $K_h(\cdot )$ is the *kernel function*, and determines the weighting. In this work we shall consider three, the first of which is the classic *Epanechnikov kernel* Epanechnikov (1969)

2.13a $$\begin{aligned} \displaystyle K_{h}(u)=\displaystyle \left\{ \begin{array}{ll} \displaystyle \frac{3}{4h}(1-u^2), & \displaystyle -1\le u<0,\\ \displaystyle 0, & \displaystyle 0 \le u. \end{array}\right. \end{aligned}$$

Since this kernel is supported on the half-interval $(t_j-h,t_j)$ it is a *left-sided* kernel, and amounts to using only historical information. We shall also consider *right-sided*

2.13b $$\begin{aligned} \displaystyle K_{h}(u)=\displaystyle \left\{ \begin{array}{ll} \displaystyle \frac{3}{4h}(1-u^2), & \displaystyle 0 \le u < 1,\\ \displaystyle 0, & \displaystyle \textrm{otherwise}. \end{array}\right. \end{aligned}$$

and *centered*

2.13c $$\begin{aligned} \displaystyle K_{h}(u)=\displaystyle \left\{ \begin{array}{ll} \displaystyle \frac{3}{4h}(1-u^2), & \displaystyle -1\le u \le 1,\\ \displaystyle 0, & \displaystyle \textrm{otherwise}. \end{array}\right. \end{aligned}$$

versions of (2.13a). Then the solution to (2.12) is found by taking the derivative of the right-hand side of (2.12) with respect to $a_0(i)$ and $a_1(i)$, setting these expressions equal to zero, and solving the resulting system to obtain

2.14a $$\begin{aligned}&\widehat{a}_0(i)=\frac{\sum _{j=1}^N(\Delta X_j/\Delta t-\widehat{a}_1(i)X_j)K_{h}(i,j)}{S(i)},\end{aligned}$$

2.14b $$\begin{aligned}&\widehat{a}_1(i)=\frac{S\sum _{j=1}^N\frac{\Delta X_j}{\Delta t} X_j K_{h}(i,j)-\left[ \sum _{j=1}^N\frac{X_j}{\Delta t}K_{h}(i,j) \right] \left( \sum _{j=1}^N K_{h}(i,j)X_j\right) }{S(i)\sum _{j=1}^NX_j^2K_{h}(i,j)-\left( \sum _{j=1}^NX_jK_{h}(i,j)\right) ^2}. \end{aligned}$$

In (2.14) we have introduced the notation

2.15a $$\begin{aligned}&K_{h}(i,j)=K_{h}\left( \frac{t_i-t_j}{h}\right) ,\end{aligned}$$

2.15b $$\begin{aligned}&S(i)=\sum _{j=1}^NK_{h}\left( \frac{t_i-t_j}{h}\right) . \end{aligned}$$

Equation (2.20) gives us an estimate for the drift coefficients, so we now turn our attention to estimating the diffusion coefficient. We begin by observing that equation (2.1a) leads to the following model for the residual

2.16 $$\begin{aligned} \Delta X_i-[\widehat{a}_0(i)+\widehat{a}_1(i)]\Delta t\approx b(t_i)\Delta W_i. \end{aligned}$$

Next we normalize the left-hand side of (2.16) and introduce the variable

2.17 $$\begin{aligned} \widehat{E}_i:=\frac{\Delta X_i-[\widehat{a}_0(i)+\widehat{a}_1(i)]\Delta t}{\sqrt{\Delta t}}. \end{aligned}$$

From (2.16) and the fact that $\Delta W_i$ has zero mean and variance $\sqrt{\Delta t}$, it follows that $\widehat{E}_i\sim {\mathcal {N}}(0, b^2(t_i))$ and that its conditional density given information up to time $t_i$ is

2.18 $$\begin{aligned} [2\pi b^2(i)]^{-1/2}\exp \left( -\frac{\widehat{E}^2_i}{2b(t_i)^2}\right) . \end{aligned}$$

Equation (2.18) suggests the local weighted log-likelihood function

2.19 $$\begin{aligned} L(b(t_i),t_i)=-\frac{1}{2}\sum _{j=1}^NK_{h}(i,j)\left( \log b^2(t_i)+\frac{\widehat{E}_j^2}{b(i)^2}\right) , \end{aligned}$$

and taking $\widehat{b}(i)$ to be the argument maximum of (2.19) we find

2.20 $$\begin{aligned} \widehat{b}(i)=\sqrt{\frac{\sum _{j=1}^NK_{h}(i,j)\widehat{E}_j^2}{\sum _{j=1}^NS(j)}}. \end{aligned}$$

With the estimated drift (2.14) and diffusion (2.20) coefficients, the estimated reconstruction $Y_i$ to the original signal $X_{t_i}$ is given by

2.21a $$\begin{aligned}&\Delta Y_{i}=[\widehat{a}_0(i)+\widehat{a}_1(i)]\Delta t_i+\widehat{b}(i)\Delta W_{i},\end{aligned}$$

2.21b $$\begin{aligned}&Y_0=X_t|_{t=0}. \end{aligned}$$

The deterministic component of the estimated signal is then given by

2.22a $$\begin{aligned}&\Delta Y^{(\textrm{d})}_{i}=[\widehat{a}_0(i)+\widehat{a}_1(i)]\Delta t,\end{aligned}$$

2.22b $$\begin{aligned}&Y^{(\textrm{d})}_0=X_t|_{t=0}, \end{aligned}$$

for $i=0,\ \ldots ,\ M-1$, and the stochastic component through the formula

2.23 $$\begin{aligned} \Delta Y^{(\textrm{w})}_{i}=\widehat{b}(i)\Delta W_{i}, \end{aligned}$$

for $i=1,\ \ldots ,\ M$. In writing (2.22) and (2.23) we have let $Y^{(\textrm{d})}$ and $Y^{(\textrm{w})}$ denote the deterministic and stochastic components of the estimated signal respectively. So given a measurement $X_t$ from a Bio-FET experiment, we can reconstruct it through the formula (2.21) and extract its deterministic (2.22) and stochastic (2.23) components. The estimated deterministic component (2.22) gives us an approximation to what in the original measurement corresponds to true signal, and (2.23) gives us an approximation to what in the original measurement corresponds to noise. These approximations depend on the estimated coefficients (2.14) and (2.20), which in turn depend upon the kernel function and the bandwidth parameter. It is clear that some choices of the bandwidth parameter will result in more accurate reconstructions of the signal than others. If the bandwidth parameter is taken to be too large, then our approximation to the coefficients (2.14), (2.20) will be poor and so will the reconstruction $Y_i$. If the bandwidth parameter is taken to be too small, then our approximation to the coefficients will (2.14), (2.20) also be poor and so will the reconstruction $Y_i$.

## Optimal bandwidth for a Bio-FET measurement

To find the optimal bandwidth parameter, we need to introduce a notion of optimality. In the present work we consider three, the first of which is the root-mean-square error (RMSE)

3.1 $$\begin{aligned} e_{RMSE} = \sqrt{\frac{1}{M+1}\sum _{i=0}^M A_i^2}, \end{aligned}$$

where

3.2 $$\begin{aligned} A_i = |X_{t_i}-Y_i(h)|. \end{aligned}$$

We will also consider the mean average error (MAE)

3.3 $$\begin{aligned} \mu = \frac{1}{M+1}\sum _{i=0}^M A_i, \end{aligned}$$

and the standard deviation ($\textrm{STD}_X$)

3.4 $$\begin{aligned} \sigma = \sqrt{\frac{1}{M}\sum _{i=0}^M(A_i-\mu )^2} . \end{aligned}$$

For each of the signals considered herein the time step $\Delta t$ is fixed, and *k* is a free parameter that may be chosen at our liberty when applying stochastic regression. Since for each signal $\Delta t$ is fixed and *k* is free, the bandwidth parameter (2.10) is a function of *k*. So to optimize (3.1)–(3.4) we take of these optimality measures to be functions of *k*, and we also report their respective argument minimums in terms of $k^*$ rather than $h^*=\Delta t\, k^*$.

To optimize (3.1)–(3.4), an exhaustive search was performed over all integral values of *k* in the range

3.5 $$\begin{aligned} 1<k< \lfloor M/2 \rfloor . \end{aligned}$$

In many cases, physically reasonable behavior of our stochastic regression algorithm may require a bandwidth parameter that is not too small. For the bandwidth parameter can be thought of as the degree of smoothing that is applied to the signal, and many signals may require a minimum amount of smoothing before physically reasonable results are produced. Signals such as the one shown in Fig. 2 may have too much variation within the window (2.9) to obtain a reasonable approximation for the coefficients $a_0(t)$, $a_1(t)$, and *b*(*t*) when *k* is too small. On the other hand *k* cannot be too large, for this would violate our assumption that the coefficients are well-approximated by constants over the window (2.9). So we formally restricted ourselves to values of *k* within (3.5) that were large enough to produce a physically reasonable approximation to the signal, and small enough to not violate our assumption that the coefficients are well-approximated by constants over the window (2.9). This choice was confirmed by the fact that, for all kernel and optimality measure pairings, the global minimum over (3.5) was consistently and uniformly within our restriction.

**Fig. 3:**
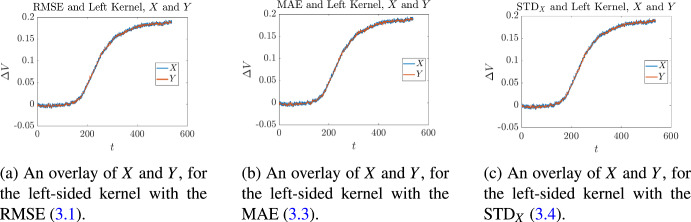
Overlay of the signal *X* and its optimal reconstructions *Y* for the left-sided kernel, for each of the objectives (3.1)–(3.4)

**Fig. 4:**
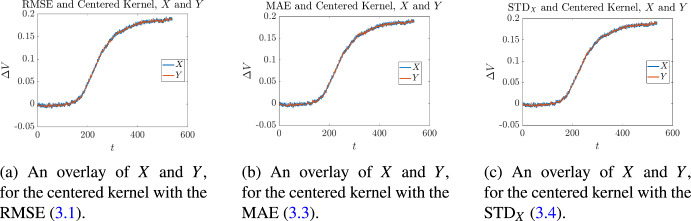
Overlay of the signal *X* and its optimal reconstructions *Y* for the centered kernel, for each of the objectives (3.1)–(3.4)

**Fig. 5:**
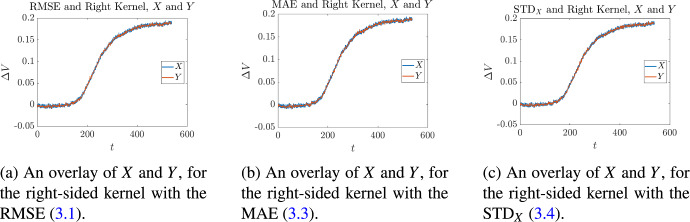
Overlay of the signal *X* and its optimal reconstructions *Y* for the right-sided kernel, for each of the objectives (3.1)–(3.4)

This procedure was applied to find optimal reconstructions of the signal shown in Fig. 2, and the associated optimal bandwidth values. The results are tabulated in Table 1, and shown in Figs. 3–5. From the latter, it is clear that all of the pairings between each kernel and each optimality measure produce signals that agree very well with the original signal *X*. The data in Table 1 shows that the curves match up quantitatively as well as qualitatively. We see that for each of the optimality measures, the centered kernel performs the best. This is to be expected, since centered kernels use information from both sides of the window (2.9), while its left and right-sided counterparts use information from only one side.

**Table 1:** Optimal bandwidth parameters and associated objective function values for the signal shown in Figs. 3 to 5

Kernel	RMSE		MAE		$\textrm{STD}_X$	
	$k^*$	(3.1)	$k^*$	(3.3)	$k^*$	(3.4)
Left Kernel	12	${1.7611 \times 10^{-3}}$	12	${1.3467\times 10^{-3}}$	12	${1.1348\times 10^{-3}}$
Centered Kernel	4	${1.7190\times 10^{-3}}$	4	${1.3038\times 10^{-3}}$	4	${1.1204\times 10^{-3}}$
Right Kernel	7	1.7686	6	${1.3259\times 10^{-3}}$	12	${1.1705\times 10^{-3}}$

## Cross validation

Although the signal studied in Sect. 3 came from a single instrument, in practice it is not uncommon to perform many experiments with instruments that have different dimensions. One important way in which these instruments may differ from each other is with respect to their *aspect ratio* $\epsilon$, the ratio of the solution-well’s height to its diameter. To examine the performance of our algorithm when applied to instruments with different aspect ratios, cross validation was conducted by first selecting ten numbers in a uniformly random manner between the minimum $\epsilon _{\mathrm {\min }}=0.1250$ and maximum $\epsilon _{\max }=5$ aspect ratios, shown in Table 2. Then using the physical model presented in Evans et al. (2020) ten curves were created, one for each of the aspect ratios. Normally distributed noise, with mean and variance equal to that of the estimated stochastic component of the signal analyzed in Sect. 3, was added to each of the curves. This resulted in ten synthetic measurements, each corresponding to an instrument with a different aspect ratio. By using the model presented in Evans et al. (2020) to create these synthetic measurements, the time and expense of creating ten different instruments and performing a different experiment with each of them was obviated.

**Table 2:** Aspect ratios used for cross validation

*i*	1	2	3	4	5	6	7	8	9	10
$\varepsilon _i$	4.0968	4.5407	0.74406	4.5777	3.2078	0.60051	1.4827	2.7910	4.7928	4.8288

Cross validation on these measurements was then performed in the following manner. Let us fix our attention on a single measurement corresponding to one of the $\epsilon _i$, given in Table 2. For each of the kernels given in (2.5), optimal values of (3.1), (3.3), and (3.4) were found as described in Sect. 3. For each optimality measure this resulted in an optimal value $k_i^*$, for every kernel. Then for each of the values of $k_i^*$, the average of all the *other* aspect ratios was calculated

4.1 $$\begin{aligned} {\overline{k}}_i= \frac{1}{9}\sum _{\begin{array}{c} j= 1, \\ j\not =i \end{array}}^{10} k_j^*. \end{aligned}$$

and the absolute

4.2 $$\begin{aligned} |k_i^*-{\overline{k}}_i| \end{aligned}$$

and relative

**Fig. 6:**
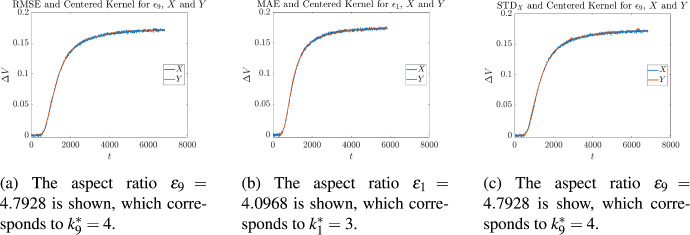
Three graphs corresponding to the optimal values of (3.1), (3.3), and (3.4), in the case of the centered kernel. In each graph there is an overlay of a synthetic measurement *X* and its approximation *Y*

**Fig. 7:**
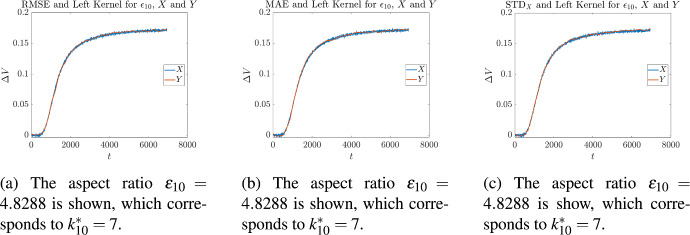
Three graphs corresponding to the optimal values of (3.1), (3.3), and (3.4), in the case of the left-sided kernel. In each graph there is an overlay of a synthetic measurement *X* and its approximation *Y*

**Fig. 8:**
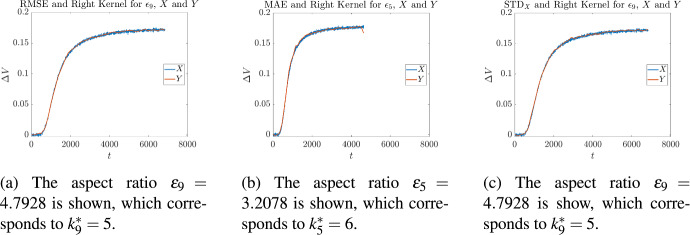
Three graphs corresponding to the optimal values of (3.1), (3.3), and (3.4), in the case of the right-sided kernel. In each graph there is an overlay of a synthetic measurement *X* and its approximation *Y*

4.3 $$\begin{aligned} \frac{|k_i^*-{\overline{k}}_i|}{k_i^*} \end{aligned}$$

differences were measured. The results for the centered kernel are given in Tables 3 to 5, the results for the left-sided kernel are given in Tables 6 to 8, and the results for the right-sided kernel are given in Tables 9 to 11. Illustrative overlays of synthetic measurements and the curves obtained by applying stochastic regression are shown in Figs. 6 to 8. The curves depicted are those for which the minimum error measure was achieved, for a fixed kernel. So for example, since $\epsilon _9$ corresponds to the instrument with the smallest objective function value in Table 3, this is the curve that has been shown in Fig. 6a. When inspecting these figures one may notice that some curves reach steady state faster than others; for example, in Figs. 6a and 6b the terminal times are approximately $T={7000}$ and $T={6000}$ respectively. This is because instruments that have different aspect ratios differ in the time taken to achieve equilibrium. In general, those with smaller values of $\epsilon$ tend to reach steady state quicker because the initial injection of chemical reactant is physically closer to the biochemical gate, which is exactly what we see in Figs. 6a and 6b. The latter corresponds to an instrument with a smaller aspect ratio than the former, and so reaches equilibrium faster.

**Table 3:** Cross validation of stochastic regression with respect to aspect ratios, when using a centered kernel and the objective (3.1)

Aspect Ratio	Objective (3.1)		Cross Validation		
$\varepsilon _i$	$k^*_i$	$e_{\textrm{RMSE}}$	${\overline{k}}_i$	$|{\overline{k}}_i-k^*_i|$	$\displaystyle \frac{|{\overline{k}}_i-k^*_i|}{k^*_i}$
4.0968	4	${1.6426 \times 10^{-3}}$	$4.{\overline{5}}$	$0.{\overline{5}}$	$0.13{\overline{8}}$
4.5407	4	${1.6741\times 10^{-3}}$	$4.{\overline{5}}$	$0.{\overline{5}}$	$0.13{\overline{8}}$
0.74406	4	${1.6693\times 10^{-3}}$	$4.{\overline{5}}$	$0.{\overline{5}}$	$0.13{\overline{8}}$
4.5777	4	${1.6578\times 10^{-3}}$	$4.{\overline{5}}$	$0.{\overline{5}}$	$0.13{\overline{8}}$
3.2078	4	${1.6609\times 10^{-3}}$	$4.{\overline{5}}$	$0.{\overline{5}}$	$0.13{\overline{8}}$
0.60051	4	${1.6612\times 10^{-3}}$	$4.{\overline{5}}$	$0.{\overline{5}}$	$0.13{\overline{8}}$
1.4827	6	${1.6949\times 10^{-3}}$	$4.{\overline{3}}$	$1.{\overline{6}}$	$0.2{\overline{7}}$
2.7910	6	${1.7784\times 10^{-3}}$	$4.{\overline{3}}$	$1.{\overline{6}}$	$0.2{\overline{7}}$
4.7928	4	${1.6292\times 10^{-3}}$	$4.{\overline{5}}$	$0.{\overline{5}}$	$0.13{\overline{8}}$
4.8288	5	${1.7217\times 10^{-3}}$	$4.{\overline{4}}$	$0.{\overline{5}}$	$0.{\overline{1}}$

**Table 4:** Cross validation of stochastic regression with respect to aspect ratios, when using a centered kernel and the objective (3.3)

Aspect Ratio	Objective (3.3)		Cross Validation		
$\varepsilon _i$	$k^*_i$	$\mu$	${\overline{k}}_i$	$|{\overline{k}}_i-k^*_i|$	$\displaystyle \frac{|{\overline{k}}_i-k^*_i|}{k^*_i}$
4.0968	3	${1.2461\times 10^{-3}}$	$4.{\overline{1}}$	$1.{\overline{1}}$	$0.{\overline{370}}$
4.5407	4	${1.2884\times 10^{-3}}$	4	0	0
0.74406	4	${1.2872\times 10^{-3}}$	4	0	0
4.5777	4	${1.2872\times 10^{-3}}$	4	0	0
3.2078	4	${1.2734\times 10^{-3}}$	4	0	0
0.60051	4	${1.2601\times 10^{-3}}$	4	0	0
1.4827	4	${1.3099\times 10^{-3}}$	4	0	0
2.7910	3	${1.3575\times 10^{-3}}$	$4.{\overline{1}}$	$1.{\overline{1}}$	$0.{\overline{370}}$
4.7928	4	${1.2648\times 10^{-3}}$	4	0	0
4.8288	6	${1.3314\times 10^{-3}}$	$3.{\overline{7}}$	$2.{\overline{2}}$	$0.{\overline{370}}$

**Table 5:** Cross validation of stochastic regression with respect to aspect ratios, when using a centered kernel and the objective (3.4)

Aspect Ratio	Objective (3.4)		Cross Validation		
$\varepsilon _i$	$k^*_i$	$\sigma$	${\overline{k}}_i$	$|{\overline{k}}_i-k^*_i|$	$\displaystyle \frac{|{\overline{k}}_i-k^*_i|}{k^*_i}$
4.0968	4	${1.0485\times 10^{-3}}$	$5.{\overline{2}}$	$1.{\overline{2}}$	$0.30{\overline{5}}$
4.5407	5	${1.0578\times 10^{-3}}$	$5.{\overline{1}}$	$0.{\overline{1}}$	$0.0{\overline{2}}$
0.74406	4	${1.0631\times 10^{-3}}$	$5.{\overline{2}}$	$1.{\overline{2}}$	$0.30{\overline{5}}$
4.5777	7	${1.0399\times 10^{-3}}$	$4.{\overline{8}}$	$2.{\overline{1}}$	0.30159
3.2078	5	${1.0661\times 10^{-3}}$	$5.{\overline{1}}$	$0.{\overline{1}}$	$0.0{\overline{2}}$
0.60051	5	${1.0413\times 10^{-3}}$	$5.{\overline{1}}$	$0.{\overline{1}}$	$0.0{\overline{2}}$
1.4827	6	${1.0731\times 10^{-3}}$	5	1	$0.1{\overline{6}}$
2.7910	6	${1.0976\times 10^{-3}}$	5	1	$0.1{\overline{6}}$
4.7928	4	${1.0271\times 10^{-3}}$	$5.{\overline{2}}$	$1.{\overline{2}}$	$0.30{\overline{5}}$
4.8288	5	${1.0755\times 10^{-3}}$	$5.{\overline{1}}$	$0.{\overline{1}}$	$0.0{\overline{2}}$

A consistent theme across all kernels and all optimality measures is that the optimal value $k_i^*$ is within a factor two to three of the predicted value ${\overline{k}}_i$. This shows that there is not large variation of $k_i^*$ between instruments with different aspect ratios, so that $k_i^*$ for one instrument will be very close to that of another. There are exceptions; for example, the results for the left-sided kernel show that $|{\overline{k}}_i-k_i^*|$ is typically around six for the aspect ratio $\epsilon _6=0.60051$. An examination of the results shown in Tables 3 to 11 reveals that this is indeed an exception, and that our stochastic regression algorithm enjoys remarkable consistency across Bio-FETs with different aspect ratios.

The results in Tables 3 to 11 may be used to guide the choice of kernel in our algorithm. For all aspect ratios and each optimality measure, the centered kernel always has a value of $k_i^*$ less than or equal to that of either its left or right-sided counterparts. Smaller values of $k_i^*$ correspond to smaller averaging windows, which increases the accuracy with which the coefficients $\widehat{a}_0(i)$, $\widehat{a}_1(i)$, and $\widehat{b}(i)$ are estimated. Moreover, for every optimality measure, the centered kernel always has the smallest objective function value. This suggests that using a centered kernel may recover the true signal with the greatest accuracy.

It is also the case that the centered kernel is no less consistent than the others, when consistency is measured in terms of the absolute difference $|k_i^*-{\overline{k}}_i|$. This is the appropriate measure of consistency, rather than the relative difference $|k_i^*-{\overline{k}}_i|/k_i^*$. To see why this is the case, let us consider the absolute and relative differences corresponding to $\epsilon _3=0.74406$ for for the $\textrm{STD}_X$, in the case of both the centered kernel and the left-sided kernel. The absolute difference for the former is $|5.{\overline{2}}-4|=1.{\overline{2}}$ while the absolute difference for the latter is $|9.{\overline{4}}-8|=1.{\overline{4}}$, suggesting that the centered kernel is superior in this case. However the relative difference for the centered kernel is $|5.{\overline{2}}-4|/4=0.30{\overline{5}}$ while the relative difference for the left-sided kernel is $|9.{\overline{4}}-8|/8=0.180{\overline{5}}$, suggesting that left-sided kernel is superior. The slightly larger magnitude of $k_3^*$ for the left-sided kernel tips the scales in its favor when considering the relative difference. This is misleading, since in this context $k_3^*$ for the left-sided kernel is not significantly larger than $k_3^*$ for the centered kernel. For this reason the absolute difference is the appropriate measure of performance. For the RMSE, since there are seven aspect ratios for which the absolute difference of the centered kernel is less than that of the right-sided kernel, we can say that the centered kernel performs better than the left-sided kernel seven times and is superior when measured in the RMSE. The centered kernel performs better than the right-sided kernel five times, so the two are equal in the RMSE. Similar analyses can be given for the MAE and standard deviation. In the case of the former, the centered outperforms the left-sided eight times and the right-sided seven times. It is worth noting that for the MAE, *the relative difference of the centered kernel is zero for seven of the ten aspect ratios*. With respect to standard deviation, the centered kernel outperforms the left-sided kernel six times and the right-sided kernel five times. So there is no case in which the centered kernel performs worse, and it often performs better. In this sense the centered kernel is no less consistent than the others and it is often more so. It is for these reasons that we prefer the centered kernel over the others.

**Table 6:** Cross validation of stochastic regression with respect to aspect ratios, when using a left-sided kernel and the objective (3.1)

Aspect Ratio	Objective (3.1)		Cross Validation		
$\varepsilon _i$	$k^*_i$	$e_{\textrm{RMSE}}$	${\overline{k}}_i$	$|{\overline{k}}_i-k^*_i|$	$\displaystyle \frac{|{\overline{k}}_i-k^*_i|}{k^*_i}$
4.0968	10	${2.0503\times 10^{-3}}$	$9.{\overline{2}}$	$0.{\overline{7}}$	$0.0{\overline{7}}$
4.5407	8	${1.9183\times 10^{-3}}$	$9.{\overline{4}}$	$1.{\overline{4}}$	$0.180{\overline{5}}$
0.74406	8	${1.9474\times 10^{-3}}$	$9.{\overline{4}}$	$1.{\overline{4}}$	$0.180{\overline{5}}$
4.5777	9	${1.9504\times 10^{-3}}$	$9.{\overline{3}}$	$0.{\overline{3}}$	$0.{\overline{370}}$
3.2078	10	${2.0022\times 10^{-3}}$	$9.{\overline{2}}$	$0.{\overline{7}}$	$0.0{\overline{7}}$
0.60051	15	${2.2262\times 10^{-3}}$	$8.{\overline{6}}$	$6.{\overline{3}}$	$0.4{\overline{2}}$
1.4827	10	${2.2117\times 10^{-3}}$	$9.{\overline{2}}$	$0.{\overline{7}}$	$0.0{\overline{7}}$
2.7910	7	${1.9821\times 10^{-3}}$	$9.{\overline{5}}$	$2.{\overline{5}}$	0.36508
4.7928	9	$1.8670 \times 10^{-3}$	$9.{\overline{3}}$	$0.{\overline{3}}$	$0.0{\overline{370}}$
4.8288	7	${1.8151\times 10^{-3}}$	$9.{\overline{5}}$	$2.{\overline{5}}$	$0.{\overline{365079}}$

**Table 7:** Cross validation of stochastic regression with respect to aspect ratios, when using a left-sided kernel and the objective (3.3)

Aspect Ratio	Objective (3.3)		Cross Validation		
$\varepsilon _i$	$k^*_i$	$\mu$	${\overline{k}}_i$	$|{\overline{k}}_i-k^*_i|$	$\displaystyle \frac{|{\overline{k}}_i-k^*_i|}{k^*_i}$
4.0968	10	${1.5403\times 10^{-3}}$	9	1	0.1
4.5407	8	${1.4915\times 10^{-3}}$	$9.{\overline{2}}$	$1.{\overline{2}}$	$0.152{\overline{7}}$
0.74406	8	${1.4491\times 10^{-3}}$	$9.{\overline{2}}$	$1.{\overline{2}}$	$0.152{\overline{7}}$
4.5777	9	${1.5026\times 10^{-3}}$	$9.{\overline{1}}$	$0.{\overline{1}}$	0.012346
3.2078	10	${1.4955\times 10^{-3}}$	9	1	0.1
0.60051	15	${1.5832\times 10^{-3}}$	$8.{\overline{4}}$	$6.{\overline{5}}$	$0.4{\overline{370}}$
1.4827	8	${1.5315\times 10^{-3}}$	$9.{\overline{2}}$	$1.{\overline{2}}$	$0.152{\overline{7}}$
2.7910	7	${1.4773\times 10^{-3}}$	$9.{\overline{3}}$	$2.{\overline{3}}$	$0.{\overline{3}}$
4.7928	9	$1.4440 \times 10^{-3}$	$9.{\overline{1}}$	$0.{\overline{1}}$	0.012346
4.8288	7	${1.3931\times 10^{-3}}$	$9.{\overline{3}}$	$2.{\overline{3}}$	$0.{\overline{3}}$

**Table 8:** Cross validation of stochastic regression with respect to aspect ratios, when using a left-sided kernel and the objective (3.4)

Aspect Ratio	Objective (3.4)		Cross Validation		
$\varepsilon _i$	$k^*_i$	$\sigma$	${\overline{k}}_i$	$|{\overline{k}}_i-k^*_i|$	$\displaystyle \frac{|{\overline{k}}_i-k^*_i|}{k^*_i}$
4.0968	10	${1.3536\times 10^{-3}}$	$9.{\overline{2}}$	$0.{\overline{7}}$	$0.0{\overline{7}}$
4.5407	8	${1.2067\times 10^{-3}}$	$9.{\overline{4}}$	$1.{\overline{4}}$	$0.180{\overline{5}}$
0.74406	8	${1.3013\times 10^{-3}}$	$9.{\overline{4}}$	$1.{\overline{4}}$	$0.180{\overline{5}}$
4.5777	9	${1.2437\times 10^{-3}}$	$9.{\overline{3}}$	$0.{\overline{3}}$	$0.{\overline{037}}$
3.2078	10	${1.3316\times 10^{-3}}$	$9.{\overline{2}}$	$0.{\overline{7}}$	$0.0{\overline{7}}$
0.60051	15	${1.5664\times 10^{-3}}$	$8.{\overline{6}}$	$6.{\overline{3}}$	$0.4{\overline{2}}$
1.4827	10	${1.5169\times 10^{-3}}$	$9.{\overline{2}}$	$0.{\overline{7}}$	$0.0{\overline{7}}$
2.7910	7	${1.3218\times 10^{-3}}$	$9.{\overline{5}}$	$2.{\overline{5}}$	0.36508
4.7928	9	${1.1837\times 10^{-3}}$	$9.{\overline{3}}$	$0.{\overline{3}}$	$0.{\overline{037}}$
4.8288	7	${1.1638\times 10^{-3}}$	$9.{\overline{5}}$	$2.{\overline{5}}$	0.36508

**Table 9:** Cross validation of stochastic regression with respect to aspect ratios, when using a right-sided kernel and the objective (3.1)

Aspect Ratio	Objective (3.1)		Cross Validation		
$\varepsilon _i$	$k^*_i$	$e_{\textrm{RMSE}}$	${\overline{k}}_i$	$|{\overline{k}}_i-k^*_i|$	$\displaystyle \frac{|{\overline{k}}_i-k^*_i|}{k^*_i}$
4.0968	7	${1.8295\times 10^{-3}}$	$6.{\overline{3}}$	$0.{\overline{6}}$	${9.5238 \times 10^{-2}}$
4.5407	7	${1.8016\times 10^{-3}}$	$6.{\overline{3}}$	$0.{\overline{6}}$	${9.5238 \times 10^{-2}}$
0.74406	7	${1.9946\times 10^{-3}}$	$6.{\overline{3}}$	$0.{\overline{6}}$	${9.5238 \times 10^{-2}}$
4.5777	7	${1.7855\times 10^{-3}}$	$6.{\overline{3}}$	$0.{\overline{6}}$	${9.5238\times 10^{-2} }$
3.2078	6	${1.7589\times 10^{-3}}$	$6.{\overline{4}}$	$0.{\overline{4}}$	$0.{\overline{074}}$
0.60051	7	${1.9269\times 10^{-3}}$	$6.{\overline{3}}$	$0.{\overline{6}}$	${9.5238\times 10^{-2}}$
1.4827	6	${1.8009\times 10^{-3}}$	$6.{\overline{4}}$	$0.{\overline{4}}$	$0.{\overline{074}}$
2.7910	6	${1.8604\times 10^{-3}}$	$6.{\overline{4}}$	$0.{\overline{4}}$	$0.{\overline{074}}$
4.7928	5	${1.7152\times 10^{-3}}$	$6.{\overline{5}}$	$1.{\overline{5}}$	$0.3{\overline{1}}$
4.8288	6	${1.7909\times 10^{-3}}$	$6.{\overline{4}}$	$0.{\overline{4}}$	$0.{\overline{074}}$

**Table 10:** Cross validation of stochastic regression with respect to aspect ratios, when using a right-sided kernel and the objective (3.3)

Aspect Ratio	Objective (3.3)		Cross Validation		
$\varepsilon _i$	$k^*_i$	$\mu$	${\overline{k}}_i$	$|{\overline{k}}_i-k^*_i|$	$\displaystyle \frac{|{\overline{k}}_i-k^*_i|}{k^*_i}$
4.0968	6	${1.3821\times 10^{-3}}$	$6.{\overline{3}}$	$0.{\overline{3}}$	$0.0{\overline{5}}$
4.5407	7	${1.3751\times 10^{-3}}$	$6.{\overline{2}}$	$0.{\overline{7}}$	$0.{\overline{1}}$
0.74406	8	${1.5024\times 10^{-3}}$	$6.{\overline{1}}$	$1.{\overline{8}}$	$0.236{\overline{1}}$
4.5777	7	${1.3938\times 10^{-3}}$	$6.{\overline{2}}$	$0.{\overline{7}}$	$0.{\overline{1}}$
3.2708	6	${1.3266\times 10^{-3}}$	$6.{\overline{3}}$	$0.{\overline{3}}$	$0.0{\overline{5}}$
0.60051	7	${1.4302\times 10^{-3}}$	$6.{\overline{2}}$	$0.{\overline{7}}$	$0.{\overline{1}}$
1.4827	6	${1.3562\times 10^{-3}}$	$6.{\overline{3}}$	$0.{\overline{3}}$	$0.0{\overline{5}}$
2.7910	5	${1.4431\times 10^{-3}}$	$6.{\overline{4}}$	$1.{\overline{4}}$	$0.2{\overline{8}}$
4.7928	5	${1.3391\times 10^{-3}}$	$6.{\overline{4}}$	$1.{\overline{4}}$	$0.2{\overline{8}}$
4.8288	6	${1.3549\times 10^{-3}}$	$6.{\overline{3}}$	$0.{\overline{3}}$	$0.0{\overline{5}}$

**Table 11:** Cross validation of stochastic regression with respect to aspect ratios, when using a right-sided kernel and the objective (3.4)

Aspect Ratio	Objective (3.4)		Cross Validation		
$\varepsilon _i$	$k^*_i$	$\sigma$	${\overline{k}}_i$	$|{\overline{k}}_i-k^*_i|$	$\displaystyle \frac{|{\overline{k}}_i-k^*_i|}{k^*_i}$
4.0968	7	${1.7763\times 10^{-3}}$	$6.{\overline{3}}$	$0.{\overline{6}}$	0.095238
4.5407	7	${1.1643\times 10^{-3}}$	$6.{\overline{3}}$	$0.{\overline{6}}$	0.095238
7.4406	7	${1.3042\times 10^{-3}}$	$6.{\overline{3}}$	$0.{\overline{6}}$	0.095238
4.5777	7	${1.1162\times 10^{-3}}$	$6.{\overline{3}}$	$0.{\overline{6}}$	0.095238
3.2078	6	${1.1552\times 10^{-3}}$	$6.{\overline{4}}$	$0.{\overline{4}}$	$0.{\overline{074}}$
0.60051	7	${1.2916\times 10^{-3}}$	$6.{\overline{3}}$	$0.{\overline{6}}$	0.095238
1.4827	6	${1.1851\times 10^{-3}}$	$6.{\overline{4}}$	$0.{\overline{4}}$	$0.{\overline{074}}$
2.7910	6	${1.1603\times 10^{-3}}$	$6.{\overline{4}}$	$0.{\overline{4}}$	$0.{\overline{074}}$
4.7928	5	${1.0721\times 10^{-3}}$	$6.{\overline{5}}$	$1.{\overline{5}}$	$0.3{\overline{1}}$
4.8288	6	${1.1715\times 10^{-3}}$	$6.{\overline{4}}$	$0.{\overline{4}}$	$0.{\overline{074}}$

## Concluding remarks

Herein we have found the optimal bandwidth parameter for stochastic regression denoising of an experimental Bio-FET measurement. This was done by performing an exhaustive search for the minimum values of (3.1), (3.3), and (3.4) over an appropriate range of values for *k*. By pairing these different optimality measures with different kernels, we found optimal decompositions of the experimental measurement into signal and noise. The centered kernel performed the best across all optimality measures. In addition, we have used cross-validation to demonstrate that optimal bandwidths are strikingly consistent across instruments with a range of different aspect ratios. Our cross-validation also suggests that the centered kernel is the most appropriate in this application, consistent with our findings from the experimental measurement.

Bio-FETs are a promising way to measure the biomarker levels, and we expect forthcoming Bio-FET measurements to lead to exciting applications of this work. In addition to Bio-FETs, we anticipate that other applications of stochastic regression are on the horizon. Biomedical technology will continue to play an increasingly important role in healthcare and the economy. Such technology often produces times series measurements like those analyzed in the present work, and we expect that optimizing stochastic regression in these specific situations will recover the most accurate reconstruction of the true signal and in so doing give us an accurate estimate of the noise. Moreover, for many instrument applications, having an esimate of the noise is useful for calibration.

## Disclaimer

This paper is an official contribution of the National Institute of Standards and Technology, and is not subject to copyright in the United States. Commercial products are identified in order to adequately specify certain procedures. In no case does such identification imply recommendation or endorsement by the National Institute of Standards and Technology, nor does it imply that the identified products are necessarily the best available for the purpose.
